# Antitumor activity of S116836, a novel tyrosine kinase inhibitor, against imatinib-resistant FIP1L1-PDGFRα-expressing cells

**DOI:** 10.18632/oncotarget.2090

**Published:** 2014-06-11

**Authors:** Yingying Shen, Xiaomei Ren, Ke Ding, Zhang Zhang, Deping Wang, Jingxuan Pan

**Affiliations:** ^1^ Department of Pathophysiology, Zhongshan School of Medicine, Sun Yat-sen University, Guangzhou, China; ^2^ Key Laboratory of Regenerative Biology and Institute of Chemical Biology, Guangzhou Institute of Biomedicine and Health, Chinese Academy of Sciences, Guangzhou, China; ^3^ State Key Laboratory of Ophthalmology, Zhongshan Ophthalmic Center, Sun Yat-sen University, Guangzhou, China; ^4^ Collaborative Innovation Center for Cancer Medicine, State Key Laboratory of Oncology in South China, Sun Yat-Sen University Cancer Center, Guangzhou, China

**Keywords:** PDGFRα, T674I, tyrosine kinase inhibitor, imatinib, resistance, S116836, apoptosis, Bim

## Abstract

The FIP1-like-1-platelet-derived growth factor receptor alpha (FIP1L1-PDGFRα) fusion oncogene is the driver factor in a subset of patients with hypereosinophilic syndrome (HES)/chronic eosinophilic leukemia (CEL). Most FIP1L1-PDGFRα-positive patients respond well to the tyrosine kinase inhibitor (TKI) imatinib. Resistance to imatinib in HES/CEL has been described mainly due to the T674I mutation in FIP1L1-PDGFRα, which is homologous to the imatinib-resistant T315I mutation in BCR-ABL. Development of novel TKIs is imperative to overcome resistance to imatinib. We synthesized S116836, a novel TKI. In this study, we evaluated the antitumor activity of S116836 in FIP1L1-PDGFRα-expressing cells. The results showed that S116836 potently inhibited PDGFRα and its downstream signaling molecules such as STAT3, AKT, and Erk1/2. S116836 effectively inhibited the growth of the WT and T674I FIP1L1-PDGFRα-expressing neoplastic cells *in vitro* and in nude mouse xenografts. Moreover, S116836 induced intrinsic pathway of apoptosis as well as the death receptor pathway, coincided with up-regulation of the proapoptotic BH3-only protein Bim-EL through the Erk1/2 pathway. In conclusion, S116836 is active against WT and T674I FIP1L1-PDGFRα-expressing cells, and may be a prospective agent for the treatment of HES/CEL.

## INTRODUCTION

The normal range of eosinophils in the peripheral blood is 3%-5% with a corresponding absolute eosinophil count of 350-500/mm^3^ [1]. When the absolute eosinophil count is >1,500/mm^3^ for more than 6 months, hypereosinophilia is diagnosed [2]. Patients with hypereosinophilia display infiltration of eosinophils in tissues resulting in organ damage. The clinical manifestations of hypereosinophilia may vary from weakness, fatigue, cough, dyspnea, to rhinitis [3]. The cause of hypereosinophilia may be primary or secondary to other diseases such as parasite infection. After excluding secondary causes of hypereosinophilia, idiopathic hypereosinophilic syndrome (HES) is diagnosed. The incidence of HES (including chronic eosinophilic leukemia, CEL) was approximately 0.036 per 100, 000 [4]. Recent study has revealed that the pathogenesis of 10%-20% HES patients is due to the gain-of-function fusion gene FIP1L1-PDGFRα formed by interstitial chromosomal deletion on 4q12 [5]. The malignant transformation of hematopoietic stem cells by FIP1L1-PDGFRα may involve activation of nuclear factor-κB pathway [6].

FIP1L1-PDGFRα-positive HES/CEL patients are highly responsive to (low-dose) imatinib with rapid and durable hematologic remissions [5,7]. In addition, antibodies such as Mepolizumab [8] and Reslizumab [9] against interleukin 5 (IL-5), and Benralizumab [10] against IL-5 receptors to block eosinophil production, activation, migration are appealing, and are undergoing clinical trials to prevent end-organ manifestations and improve the quality of life of HES/CEL patients. Corticosteroids, interferon-α, hydroxyurea and other chemotherapy are the treatment modules for the HES/CEL patients without particular molecular targets, but the response durability and short- and long-term side effects are complicated [2]. Development of targeted therapy of HES/CEL is still the most promising and imperative approach.

Imatinib mesylate (Gleevec, STI571), an inhibitor of ABL, PDGFRα, and KIT tyrosine kinases, is successfully applied to the treatment of BCR-ABL-positive chronic myelogenous leukemia (CML) [11]. However, the development of acquired resistance to imatinib has emerged, most often due to point mutations in the ATP binding sites (e.g. T674I and D842V in PDGFRα, T315I in BCR-ABL, D816V in KIT) [12-15].

Several strategies targeting T315I BCR-ABL including blockade of kinase-addiction by the third-generation of tyrosine kinase inhibitor ponatinib [16], down-modulation of BCR-ABL by inhibition of heat shock protein [17,18] were documented. Additional inhibition of the upstream or downstream molecules of BCR-ABL might significantly enhance the effect of targeting BCR-ABL [13,17-19]. These approaches may be potentially applied to targeting imatinib-resistant FIP1L1-PDGFRα. Several compounds have been reported to overcome the T674I mutant, such as nilotinib [20], EXEL-0862 [21], PKC412 (midostaurin) [22], sorafenib [15], ponatinib [23], and DCC-2036 [24] *in vitro*. Despite so, nilotinib, midostaurin and sorafenib showed limited clinical activity in T674I FIP1L1-PDGFRα-positive HES/CEL patients. Clinic efficacy of other inhibitors remains to be evaluated. Therefore, imatinib-resistance in FIP1L1-PDGFRα-positive CEL patients is still a challenge.

**Figure 1:**
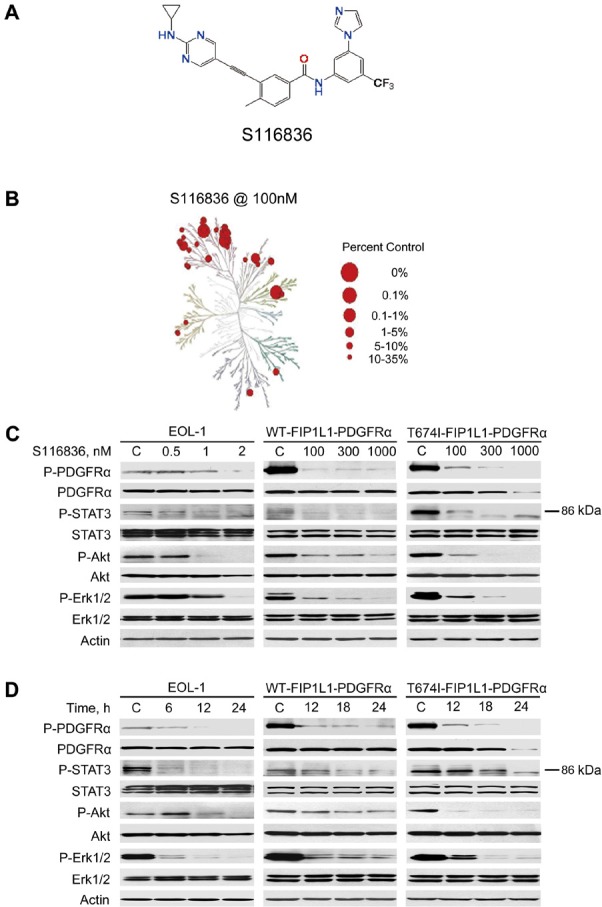
S116836 inhibits the PDGFRα kinase and its signaling A, chemical structure of compound S116836. B, S116836 holds a broad kinase activity inhibitory profiling, TREEspot^TM^ interaction maps for kinases and S116836. Kinases found to bind with S116836 are marked with red circles, where larger circles indicate higher-affinity binding. Interactions with % Ctrl @ 100 nM < 35 are shown. Mutant and lipid kinases are not represented. C, Immunoblot analysis of FIP1L1-PDGFRα-expressing cells exposed to S116836 at the indicated concentrations for 24 h. D, immunoblot analysis of FIP1L1-PDGFRα-expressing cells exposed to S116836 for the indicated durations at 2 nM (EOL-1) or 1000 nM (BaF3-WT and BaF3-T674I).

Our group took effort to develop novel tyrosine kinase inhibitors (TKIs). Small molecule GZD824 is effective against gate-keeper mutant T315I BCR-ABL [25]. In this study, we present S116836 (structure, Fig.1A) which was another novel small molecule TKI that was synthesized by rational design against T315I BCR-ABL [26]. We investigated the profile of tyrosine kinase inhibition by S116836, its activity against EOL-1 and BaF3 cells expressing wild-type FIP1L1-PDGFRα, BaF3 cells expressing FIP1L1-PDGFRα T674I mutant, and its pharmacokinetics. In addition, we studied the *in vivo* efficacy of S116836 in xenografts of FIP1L1-PDGFRα T674I cells in nude mice.

## RESULTS

### Kinase activity inhibition profiling of compound S116836

S116836 was first evaluated the kinase activity inhibition profiling. We discovered that S116836 at 100 nM potently inhibited PDGFRα tyrosine kinase activity (Table 1). In addition, S116836 showed tremendously inhibitory effect on the SRC family kinases SRC, LYN, HCK, LCK and BLK, and receptor tyrosine kinase such as FLT3, TIE2, KIT, PDGFRβ (Table 1 and Fig. 1B). Notably, S116836 not only inhibited the wild-type ABL tyrosine kinase activity, but also potently restrained the imatinib-resistant gate-keeper mutant T315I ABL tyrosine kinase activity. Taken together, S116836 is a small molecule inhibitor inhibiting multiple tyrosine kinases.

**Table 1:** Kinase inhibition profile of S116836

Kinase	% Ctrl @ 100nM
ABL1-nonphosphorylated	18
ABL1 (T315I)-nonphosphorylated	20
SRC	33
LYN	16
HCK	6.2
LCK	3.4
BLK	10
FLT3	1.8
TIE2	0.85
KIT	6.8
PDGFRα	26
PDGFRβ	3.3
PLK1	100
CDK2	99

%Ctrl @ 100 nM were determined as described in Materials and Methods. The kinase was potently inhibited by S116836 When % Ctrl @ 100 nM < 35.

### S116836 inhibits the signaling of PDGFRα

We next determined whether S116836 is capable of inhibiting FIP1L1-PDGFRα in intact cells. Toward this end, EOL-1, BaF3-WT and BaF3-T674I cells were exposed to increasing concentrations of S116836 for 24 h, the phosphorylation of FIP1L1-PDGFRα and its downstream targets were detected. The Western blotting analysis revealed that S116836 inhibited the levels of phosphorylated WT or T674I FIP1L1-PDGFRα in a dose- and time-dependent manner without significantly altering the levels of total PDGFRα (Fig. 1C and 1D).

The strikingly inhibitory effect of S116836 on FIP1L1-PDGFRα phosphorylation prompted us to examine the impact of S116836 on the phosphorylation status of downstream molecules of PDGFRα. The results demonstrated that the levels of phosphorylated STAT3, AKT, Erk1/2 were decreased after exposure to S116836, whereas no effects on total proteins were seen. These data were consistent with the downregulation of phosphorylated PDGFRα (Fig. 1C and 1D).

### S116836 inhibits growth of imatinib-sensitive and imatinib-resistant FIP1L1-PDGFRα-expressing cells

**Figure 2:**
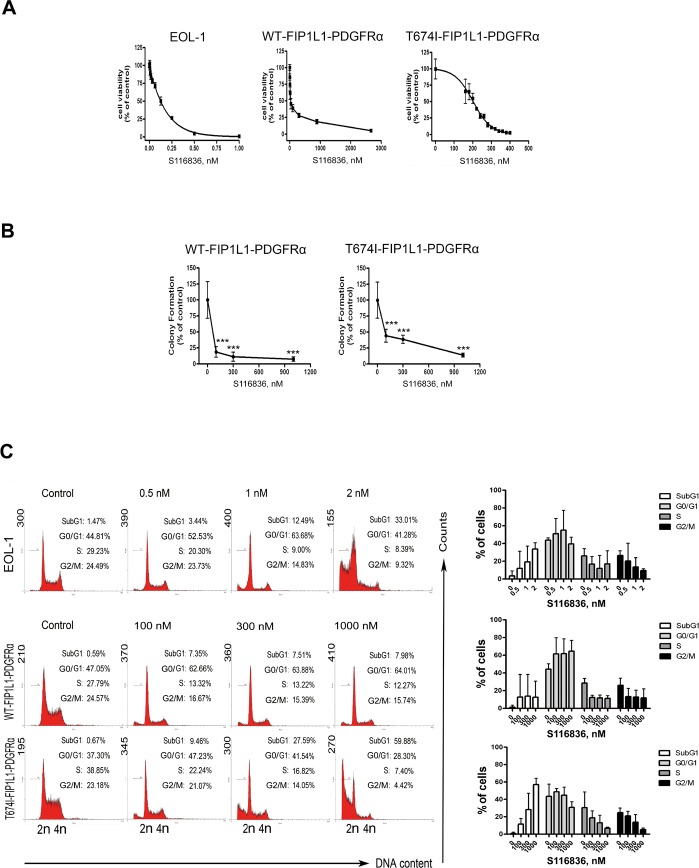
S116836 inhibits growth of FIP1L1-PDGFRα-expressing cells A,dose–response curves of FIP1L1-PDGFRα expressing cells. EOL-1, BaF3-WT and BaF3-T674I cells were cultured with various concentrations of S116836 for 72 h, cell viability was then measured by the MTS assay. Graphs show data from a representative experiment performed in triplicate; error bars represent standard deviation. B, S116836 decreases clonogenicity of FIP1L1-PDGFRα expressing BaF3 cells. BaF3-WT and BaF3-T674I cells were treated with different concentrations of S116836 for 24 h, and harvested, washed, and plated in drug-free methylcellulose culture. 7 days after incubation, the number of colonies was counted. One-way ANOVA with *post hoc* intergroup comparison by Tukey test. ***P < 0.0001. Results are presented as mean values ± 95% CI (confidence interval). C, Effect of S116836 on cell cycling in FIP1L1-PDGFRα expressing cells. EOL-1, BaF3-WT and BaF3-T674I were exposed to S116836 at the indicated concentrations for 24 h, then cells were ﬁxed and analyzed by ﬂow cytometry. Histograms show data from a representative experiment; Right, data represent the mean ± 95% CI of three independent experiments.

We next analyzed the effect of S116836 on growth in imatinib-sensitive and imatinib-resistant FIP1L1-PDGFRα-expressing cells. EOL-1, BaF3-WT and BaF3-T674I cells were treated with escalating concentrations of S116836 for 72 h, and then cell viability was ascertained by the MTS assay. S116836 was capable of inhibiting the growth of all three cell lines with an inhibitory concentration at IC_50_ of 0.2 nM, 26.9 nM, 198.8 nM, respectively (Fig. 2A).

Because clonogenicity is a better indicator of the ability of long-term proliferation in malignant tumor cells, we did the test using methylcellulose in BaF3-WT and BaF3-T674I cells. Both lines of cells were exposed to various concentrations of S116836 for 24 h, and then plated in methylcellulose cultures without S116836. S116836 significantly inhibited the surviving clonogenic BaF3-WT or BaF3-T674I cells in a dose-dependent manner (Fig. 2B).

We also investigated whether S116836 affected the cell cycle distribution. EOL-1, BaF3-WT, BaF3-T674I cells were treated with various concentrations of S116836 for 24 h, and then analyzed their DNA content by using flow cytometry. The sub-G_1_ populations were remarkably increased, suggesting that S116836 induced apoptosis (Fig. 2C).

### S116836 induces apoptosis in imatinib-sensitive and imatinib-resistant FIP1L1-PDGFRα-expressing cells

**Figure 3:**
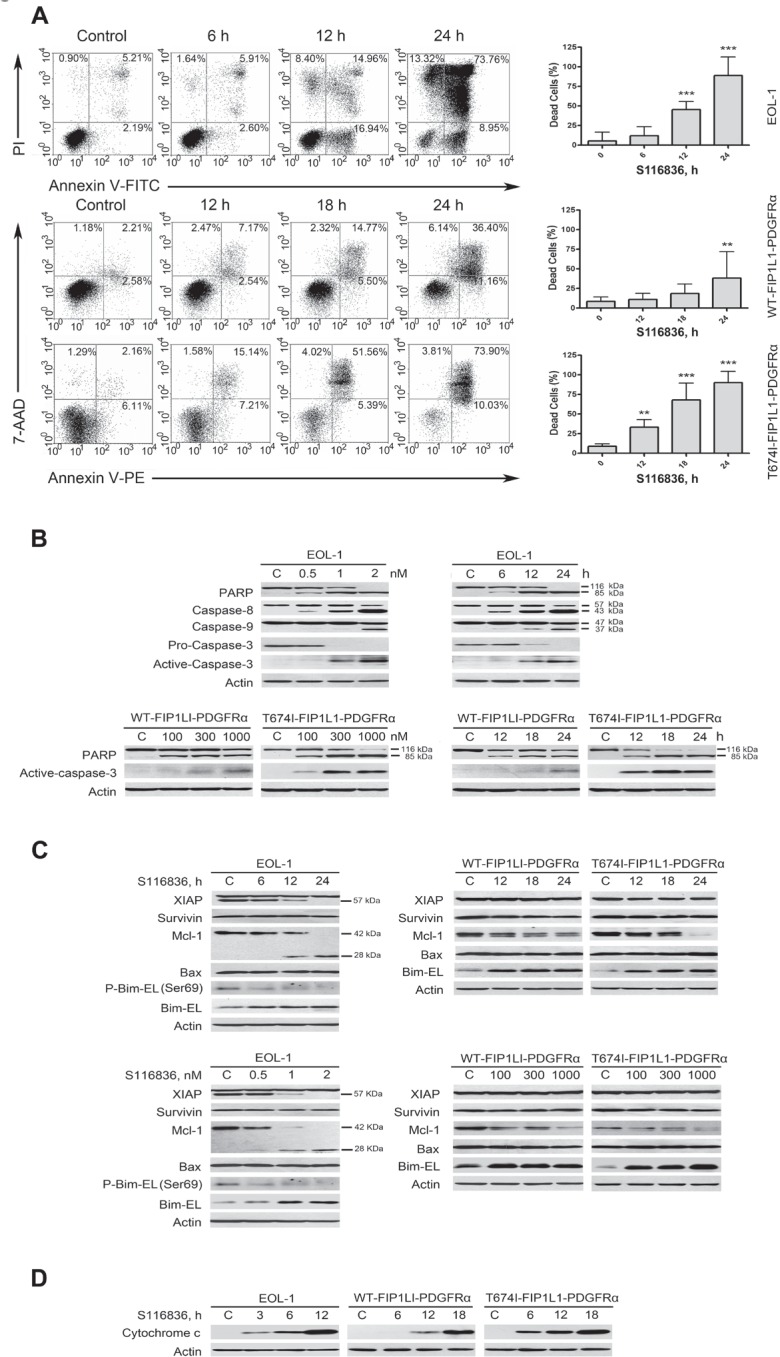
S116836 induces apoptosis in FIP1L1-PDGFRα-expressing cells A, Cells were exposed to S116836 for the indicated durations with 2 nM (EOL-1) and 1000 nM (BaF3-WT and BaF3-T674I), apoptotic cells were evaluated with ﬂow cytometer after staining with Annexin V-FITC/PI or AnnexinV-PE/7-AAD staining. *Left*, representative graphs from three independent experiments; *right*, statistical charts, vertical axis represents the sum of all dead cells except the left lower quadrant. Columns, mean; bars, 95% CI. One-way ANOVA: *post hoc* comparisons, Tukey's test. B, Concentration- and time-dependent cleavage of PARP and caspase-3 was triggered by S116836. C, Expression of apoptosis-related proteins was analyzed by Western blotting analysis. D, S116836 induced release of cytochrome c. Levels of cytochrome c in the cytosolic extracts were detected by Western blotting analysis. The concentrations of S116836 were same as (A).

The proapoptotic effect of S116836 was confirmed by ﬂow cytometry after dual staining of AnnexinV-FITC/PI or Annexin V-PE/7-AAD. S116836 induced apoptosis in EOL-1, BaF3-WT and BaF3-T674I cells in a time-dependent manner (Fig. 3A). We next reasoned that the expression of PARP, caspase-8, -9 and -3 might be affected by incubation with S116836. Indeed, S116836 induced a dose- and time-dependent specific cleavage of PARP, caspase-8, -9 and -3, which are all hallmarks of apoptosis (Fig. 3B).

To clarify the mechanism of apoptosis induced by S116836, we examined the expression of other apoptosis-related proteins with Western blotting analysis. The results revealed no change in expression of XIAP, Survivin, Bax but a substantial increase in Bim-EL level and a decrease in Mcl-1 after treatment with S116836, and both proteins changed in a time- and dose-dependent manner (Fig. 3C).

The release of cytochrome c from mitochondria into the cytosol is a marker of the intrinsic pathway of apoptosis [27]. To clarify the involvement of the apoptosis pathway triggered by S116836, we treated EOL-1, BaF3-WT and BaF3-T674I cells for different durations and then determined cytochrome c in the cytosolic fractions by Western blotting analysis. Cytochrome c was elevated in the cytosol in the cells treated with S116836 (Fig. 3D). These results indicated that S116836 induced intrinsic (mitochondrial) pathway of apoptosis.

### Up-regulation of Bim-EL is a critical mediator for S116836-induced apoptosis

**Figure 4:**
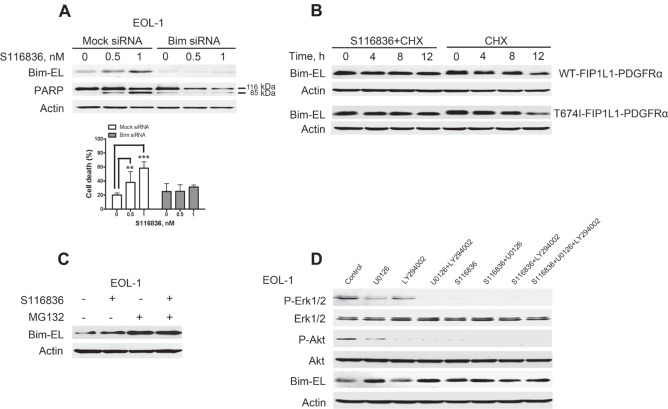
S116836 induced upregulation of Bim-EL predominantly via the Erk1/2 pathway in FIP1L1-PDGFRα- expressing cells A, knockdown of Bim expression led to attenuation of apoptosis. EOL-1 cells were transfected with human Bim siRNA or nonspeciﬁc control pool (mock siRNA), followed by treatment with S116836 at the indicated concentrations for 24 h prior to subject of Western blotting analysis (*top*) and cell death assay (trypan blue exclusion, *bottom*). Mean ± 95% CI of three independent experiments. One-way ANOVA, *post hoc* intergroup comparisons, Tukey's test. B, S116836 decelerated Bim-EL turnover. BaF3-WT and BaF3-T674I cells were incubated in the presence of S116836 100 nM for 2 h, followed by treatment with 100 μg/ml of CHX. The cells were then harvested at the indicated time points. Western blotting analysis was employed to monitor Bim-EL levels. C, Western blotting analysis in EOL-1 cells incubated with 1 nM S116836 for 24 h in the presence (plus pretreatment for 2 h) or absence of 500 nM MG132. D, Bim-EL protein levels are regulated by the Erk1/2 signaling pathway. After pre-treated with a MEK inhibitor (U0126, 10 μM) or a PI3K inhibitor (LY294002, 10 μM) for 60 minutes, EOL-1 cells were exposed to 2 nM S116836 for 12 h. Cell lysates were analyzed by immunoblotting using the indicated antibodies.

The proapoptotic BH3-only protein Bim was essential for imatinib-induced apoptosis in KIT-dependent gastrointestinal stromal tumor cells [28] and erlotinib-induced apoptosis in EGFR mutant lung cancer cells [29]. Given that Bim expression was significantly increased after S116836 treatment (Fig. 3C), we reasoned that the induction of Bim might also be a critical mediator for S116836-induced apoptosis in FIP1L1-PDGFRα expressing cells. To test this hypothesis, we evaluated the impact of silencing Bim on the sensitivity of EOL-1 cells to S116836. Transfection of siRNA duplexes against Bim in EOL-1 provoked sufficient knockdown of Bim when measured with Western blotting analysis (Fig. 4A, *top*). Bim knockdown remarkably attenuated S116836-induced apoptosis when compared with the siRNA control, as analyzed by immunoblotting for PARP cleavage (Fig. 4A, *top*) and trypan blue exclusion assay, suggesting the induction of Bim is a critical for S116836-induced apoptosis in FIP1L1-PDGFRα expressing cells (Fig. 4A, *bottom*). Because the Bim-extra long (EL) isoform Bim-EL but not Bim-long (L) and Bim-short (S) is believed the major isoform in apoptosis [30,31], the Bim- EL isoform was paid attention in subsequent experiments.

### Up-regulation of Bim-EL by S116836 is predominantly via the Erk1/2 pathway

Bim is regulated at levels of transcriptional, protein stability, and activity in various contexts [32-34]. To explore the mechanism of S116836-mediated increase in Bim-EL, we first detect the transcription of Bim with qRT-PCR. However, no remarkable change in Bim was observed in the S116836-treated cells (data not shown). We therefore turned our attention to the posttranscriptional regulation of Bim. We employed the chase experiment to evaluate whether S116836 impact on the turnover rate of Bim protein. The BaF3-WT and BaF3-T674I cells were treated with the translation inhibitor cycloheximide (CHX) to block protein synthesis, and the levels of Bim-EL were dynamically monitored at 0, 4, 8, 12 h by immunoblotting. The levels of Bim-EL decreased progressively over time in the absence of S116836 (Fig. 4B). However, the presence of S116836 slightly attenuated the decline of Bim-EL level, suggesting that S116836 might decelerate the turnover of Bim-EL (Fig. 4B).

The ubiquitin-proteasome pathway is involved in the degradation of Bim. We thus hypothesized that the ubiquitin-proteasome pathway might be involved in the turnover of Bim-EL. To this end, EOL-1 cells were incubated with S116836 in the absence or presence of the proteasome inhibitor MG132, and Bim-EL was assessed by immunoblotting. Inhibition of proteasome by MG132 led to an elevation in Bim-EL in the absence of S116836, but MG132 combined with S116836 did not further strengthen the degree of increase in Bim-EL induced by S116836. Therefore, the up-regulation of Bim-EL might occur via the proteasome pathway (Fig. 4C).

Bim has multiple phosphorylation sites among which Ser69 that Erk1/2 mediated is critical to trigger its ubiquitin-proteasome-dependent degradation [33,35]. The inactivation of PDGFRα and its subsequent dephosphorylation of Erk1/2 by S116836 were assumed to decrease levels of the phosphorylated Bim-EL at Ser69. In deed, S116836 treatment led to a decrease in phosphorylation of Bim-EL at Ser69 (Fig. 3C).

PI3K-AKT is another pathway to phosphorylate Bim to regulate its expression [32-34]. Because PDGFRα kinase inhibition by S116836 diminished activation of both Erk and AKT pathways, we determined which pathway was the main mediator to increase expression of Bim-EL. We evaluated the levels of phospho-Erk1/2, phospho-AKT and Bim-EL in lysates from EOL-1 cells exposed to S116836 in the presence or absence of U0126 (a MEK inhibitor), LY294002 (a PI3K inhibitor), or combination of all three compounds. S116836 alone or MEK inhibitor alone was sufficient to induce an increase in Bim-EL levels, whereas PI3K inhibitor alone showed minimum effect (Fig. 4D). Combinational treatment of S116836 and LY294002 did not show additive/synergistic effects on the induction of Bim-EL in contrast with the MEK inhibitor treatment alone. The results indicated that the induction of Bim-EL by S116836 may be predominantly through the MEK-Erk1/2 pathway. Triple combination of LY294002, S116836 and U0126 did not provoke additive/synergistic effects on the induction of Bim-EL either (Fig. 4D), which did not support the involvement of AKT pathway either.

### S116836 inhibits the growth of xenografted T674I-FIP1L1-PDGFRα cells in nude mice

**Figure 5:**
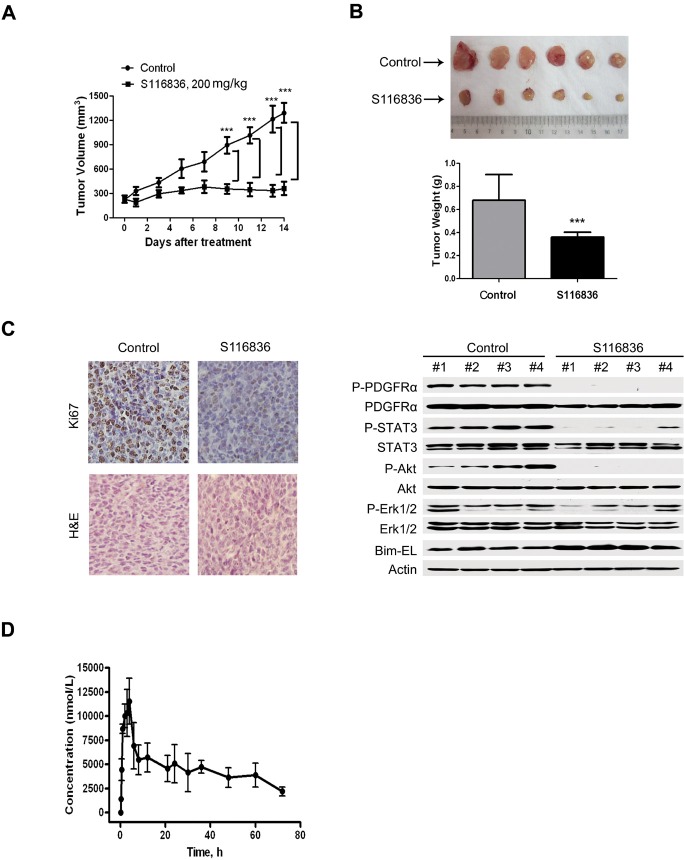
*In vivo* anti-tumor effects of S116836 A, the growth curves of subcutaneous xenografts of BaF3-T674I. Nude mice bearing BaF3-T674I xenograft tumors were treated with control (0.5% Carboxymethylcellulose) or S116836 (200 mg/kg/d, oral gavage administration) from days 5 to 19 after inoculation of BaF3-T674I cells. The estimated tumor volume was plotted versus time. Points, mean; bars, 95% CI. B, weights of tumors dissected on day 19 post-inoculation: columns, mean; bars, 95% CI. Student's t test (bottom). Photos of representative tumors are shown (*upper*). C, immunohistochemical analysis with Ki67 in xenograft tissues from mice and H & E staining (*left*). Immunoblotting of xenograft tissues from mice on day 19 after inoculation (*right*). D, Mean plasma concentration–time curve following single oral gavage administration of S116836 at 25 mg/kg. Sprague–Dawley (SD) rats (n=4).

We further evaluated the *in vivo* effect of S116836 on T674I-FIP1L1-PDGFRα cells using the nude mouse xenograft model. BaF3-T674I cells were inoculated subcutaneously in sixteen nude mice. When the size of tumor reached ~200 mm^3^ (5 days after inoculation), mice were randomized to receive treatment with 0.5% CMC (control) or S116836 (200mg/kg/d, oral gavage administration) for 14 days. We found this S116836 dosage well tolerated by preliminary experiment (data not shown). The body weights of mice were stable, with no obvious distinctions between treated and control mice. Motor activity and feeding behavior were all normal (data not shown). Collectively, inspection of morbidity and mortality didn't reflect any significant toxicity of S116836 at the dosage used. Based on the growth curve (Fig. 5A), S116836 significantly inhibited the growth of BaF3-T674I tumors. S116836-treated tumors were remarkably lower in size and weight than control tumors (Fig. 5B). Immunohistochemical analysis with anti-Ki67 antibody (to detect cell proliferation status) implied that Ki67 immunoreactivity was inhibited by S116836 treatment, which meant the cell proliferation was inhibited by S116836 (Fig. 5C, *left*). Immunoblotting of xenograft tissues from mice demonstrated that S116836 potently inhibited FIP1L1-PDGFRα and its downstream pathways, with Bim-EL expression elevated (Fig. 5C, right). All in all, these data revealed the *in vivo* anti-tumor activity of S116836 against T674I-FIP1L1-PDGFRα cells regardless of its T674I mutation status.

We also analyzed the pharmacokinetics of S116836 in SD rats. Fig. 5D is a plot of S116836 plasma concentration versus time for four SD rats, following oral gavage administration. The corresponding pharmacokinetic parameters of S116836 are summarized in Table 2, which demonstrates that they all fit into a two-compartment open model. In summary, S116836 is easy to absorb, and has a good bioavailability.

**Table 2:** Pharmacokinetics (mean ± standard deviation) of S116836 in SD rats

S116836	Dose	T1/2 (h)	AUC(0-∞)	T max	C max
PO	25 mg/kg	48.70±25.24	2516.84.38±30589.51	4.5±1.0	6205.0±731.4

Abbreviations: T1/2, half-life; AUC, area under curve; T, time; C, concentration.

## DISCUSSION

Imatinib resistance is a challenge in clinic. Clinical trials of new small molecule TKIs (e.g. nilotinib, sorafenib and midostaurin) were not ideal against T674I PDGFRα despite these drugs in vitro activity. For instance, one HES/CEL patient in blast crisis harboring T674I FIP1L1-PDGFRα showed a short response, followed by a rapid emergence of pan-resistance D842V mutation in FIP1L1-PDGFRα. In addition, mutations such as S601P [36], L629P [36] were reported to be associated with primary resistance to imatinib in FIP1L1-PDGFRα-positive patient.

In the present study, we reported that S116836 synthesized in our lab is a novel multiple TKI, and showing strikingly inhibitory effect on the activity of gate-keeper mutant T315I BCR-ABL and T674I PDGFRα at low nanomolar concentrations. S116836 potently blocked of the signaling of FIP1L1-PDGFRα and growth in the three neoplastic cells cell lines that expressed PDGFRα. In CML cells, S116836 inhibited the cell viability of KBM5 harboring BCR-ABL and KBM5-T315I in a dose-dependent fashion with IC_50_ values of 15.73-407.96 nM [26]. Similarly, S116836 potently inhibited the cell viability of HMC-1^V560G, D816V^ cells (Pan J et al., unpublished data). A single 25-mg/kg oral administration of S116836 in rats generated a maximal plasma concentration of 6205.0±731.4 (Table 2), which may be sufficient to kill imatinib-resistant cells harboring gate-keeper mutant T674I PDGFRα, T315I BCR-ABL and D816V KIT.

We have documented that S116836 induced apoptosis via the intrinsic (mitochondrial) apoptotic pathway as well as the death receptor pathway, with the increased protein levels of Bim. In addition, S116836 induced an increase in sub-G1 population, which also reflected the increase of apoptotic cells. S116836 inhibited the growth of xenografted T674I-FIP1L1-PDGFRα cells in nude mice. Taken together, S116836 could be a potential candidate for treatment of imatinib-resistant FIP1L1-PDGFRα-dependent HES/CEL.

We have demonstrated that inhibition of constitutively activated PDGFRα by S116836 triggered the up-regulation of the pro-apoptotic BH3-only protein Bim. Furthermore, knockdown of Bim by siRNA led to significant reduction in cell death, suggesting that Bim plays an important role in cell death induced by S116836 in FIP1L1-PDGFRα-positive cells. It has been previously reported that Bim was also critical for apoptosis following KIT inhibition by imatinib in GIST [28], BRAF V600E inhibition by imatinib in melanoma [37-39] and EGFR inhibition by gefitinib in non-small cell lung cancers [29,40-42]. These observations imply that Bim up-regulation may be a common mechanism for oncogenic TKI-induced apoptosis. However, knockdown of Bim didn't completely inhibit S116836-induced apoptosis in EOL-1 cells, which may result from the residual Bim protein in the siRNA transfected cells (Fig. 4A), or involvement of other regulators. One of the possible candidates is anti-apoptotic protein Mcl-1, which was observed to decrease in Fig. 3C. Mcl-1 was shown previously to be essential for EXEL-0862-induced apoptosis in FIP1L1-PDGFRα-positive cells [21]. In addition, Mcl-1 can interact with Bim and affect the activity of Bim [43,44]. Bim can be regulated at the levels of transcription and post-translational modification [45]. In this study, we have demonstrated that Bim induction occurred at the post-translational level after PDGFRα inhibition (Fig. 4B). In fact, the real-time PCR results revealed that Bim mRNA levels decreased after S116836 treatment (data not shown), which was beyond our expectation but further supported the conclusion that Bim is completed regulated at the level of post-translational modification after incubation with S116836. The ubiquitin-proteasomal degradation is among the most common ways in the post-translation modifications. With the use of proteasome inhibitor MG132, we confirmed that S116836-induced Bim up-regulation is due to the reduced degradation through proteasome pathway. Use of specific PI3K/AKT and MEK-Erk inhibitors on EOL-1 cells implies that the Erk pathway predominantly influenced Bim status downstream of PDGFRα. It has been shown that Erk1/2 kinase could phosphorylate Bim at Ser69 (Ser65 in mouse and rat) and target it for ubiquitin-proteasomal degradation [35,45]. Conversely, S116836 decreased the phosphorylated Erk1/2 and phosphorylate Bim at Ser69, hence increased dephosphorylated and deubiquitinated form of Bim. Last but not least, for the important function of Bim other strategies to increase Bim expression in FIP1L1-PDGFRα-positive cells may be a good choice to augment the potency of S116836 or prevent the drug resistance. The Bcl-2 antagonist ABT737 may also enhance S116836-induced cell killing.

In summary, our *in vitro* and *in vivo* results demonstrate that S116836 has potent activity against cells carrying wild-type or FIP1L1-PDGFRα T674I mutation. Moreover, Erk1/2-mediated Bim up-regulation is responsible for the pro-apoptotic effects of S116836 on FIP1L1-PDGFRα-positive cells. Taken together, S116836 may have clinical efﬁcacy against human neoplasms driven by FIP1L1-PDGFRα and other forms of activated PDGFRα regardless of the mutation status.

## METHODS

### Chemicals and Antibodies

S116836 (chemical structure, Fig. 1A) was rationale designed and synthesized in our lab [26]. S116836 was dissolved in DMSO at a stock concentration of 20 mM and stored in aliquots at −20°C. U0126 and LY294002 were purchased from Calbiochem (San Diego, CA). Cycloheximide (CHX) was bought from Sigma-Aldrich (St. Louis, MO). MG132 was obtained from EMD Bioscience (Billerica, MA). Antibodies against poly(adenosine diphosphate [ADP]-ribose) polymerase (PARP), pro-caspase3, X-linked inhibitor of apoptosis protein (XIAP) and cytochrome *c* were from BD Biosciences (San Jose, CA). Antibodies against phospho-PDGFRα (Y1018), phospho-Erk1/2 (T202/Y204), Erk1/2, phospho-AKT (S473), AKT, caspase-8, caspase-9, Bax and phospho-Bim (S69) were from Cell Signaling Technology (Beverly, MA). Antibodies against phospho-STAT3 (Y705), STAT3 and PDGFRα were from EMD Millipore Upstate (Billerica, MA). Anti-Mcl-1 (S-19) was from Santa Cruz Biotechnology (Santa Cruz, CA). Anti-Bim was from Stressgen Enzo Life Sciences (Plymouth Meeting, Pennsylvania). Anti-Survivin was from Novus Biologicals (Littleton, CO, USA). Anti-active-caspase-3 and Anti-β-actin were from Sigma-Aldrich (St. Louis, MO). Anti-mouse immunoglobulin G and anti-rabbit immunoglobulin G horseradish peroxidase-conjugated antibodies were purchased from Pierce Biotechnology (Los Angeles, CA, USA).

### Cell culture

The EOL-1 cell line harboring the FIP1L1-PDGFRα fusion oncogene was purchased from DMSZ (Braunschweig, Germany). BaF3 cells expressing WT or T674I FIP1L1-PDGFRα were cultured as described previously [21,23,24,46]. The presence of the FIP1L1-PDGFRα fusion in all three lines of the cells was routinely (every month) confirmed in our lab with reverse transcription PCR [7]. The T674I mutant FIP1L1-PDGFRα was verified every month by sequencing analysis of PCR products of PDGFRα exon 15. All these cells were grown in RPMI 1640 medium (Invitrogen, Guangzhou, China) supplemented with 10% fetal calf serum (FCS, Kibbutz Beit Haemek, Israel), 1 unit/mL penicillin, and 1 μg/mL streptomycin at 37°C and 5% CO_2_.

### Kinase assays

Activity of S116836 against the panel of kinases described in Table 1 and Fig. 1B was carried out at KINOMEscan, a division of Ambit Biosciences (San Diego, CA) according to the published methods [47]. The compound(s) were screened at the concentration(s) requested, and results for primary screen binding interactions are reported as ‘% Ctrl’, where lower numbers indicate stronger hits in the matrix on the table. % Ctrl @ 100 nM means the screening concentration is 100 nM. % Ctrl Calculation: [(test compound signal - positive control signal) / (negative control signal - positive control signal)] × 100; test compound = S116836, negative control = DMSO (100% Ctrl), positive control = control compound (0% Ctrl). TREEspot™ is a proprietary data visualization software tool developed by KINOMEscan. The kinase dendrogram was adapted and is reproduced with permission from Science and Cell Signaling Technology, Inc. The data were visualized by creating our own high resolution TREEspot™ interaction maps with the easy-to-use compound profile visualization tool.

### Cell viability assay

Cell viability was evaluated by MTS assay (CellTiter 96 Aqueous One Solution Cell Proliferation assay; Promega, Shanghai, China) [23,46,48,49]. Cells (2×10^5^/mL in 100 μL) plated in 96-well plates were treated with various concentration of S116836 for 72 h. The absorbance/optical density was measured with a 96-well plate reader at wavelength 490 nm after addition of MTS solution.

### Western blotting analysis

Whole lysates were prepared with RIPA buffer [48,50]. The cytosolic fraction was prepared with digitonin extraction buffer to detect the levels of cytochrome c in the cytosol [23,46,48,49].

### Apoptosis assay by flow cytometry

Apoptosis was measured with use of an annexinV-ﬂuoroisothiocyanate (for EOL-1 cells) or annexinV-phycoerythrin (for BaF3 cells expressing PDGFRα) apoptosis detection kit according to the manufacturer's instructions (Sigma-Aldrich) and analyzed with use of a FACScalibur ﬂow cytometer and CellQuestPro software as previously described [23,24,46,49]. Brieﬂy, cells were cultured in the presence of S116836, harvested and washed, and incubated in binding buffer (AnnexinV Binding Buffer, BD Pharmingen) with AnnexinV-FTIC or AnnexinV-PE for 15 min at room temperature. The cells were washed and resuspended in binding buffer. Propidium iodide (PI) or 7-amino-actinomycin (7AAD) was added just before ﬂow cytometric analysis.

### Cell cycle analysis by flow cytometry

S116836-treated cells or control cells were harvested, washed with PBS, and ﬁxed with 66% ethanol over night at −20°C. Cells were centrifuged and washed with PBS, then stained with propidium iodide (Sigma-Aldrich) and RNase in PBS solution for 1 h in the dark. Cell cycle distribution was evaluated by use of a FACScalibur ﬂow cytometer equipped with CellQuestPro software (Becton Dickinson, San Jose, CA) [23,24,46,49].

### Colony-formation assay

The colony-forming capacity of BaF3-WT and BaF3-T674I cells was analyzed by use of methylcellulose medium (Cat#03231, Stem Cell Technologies, Vancouver, Canada) according to the manufacturer's instructions. S116836 was added to the initial cultures at 100 to 1000 nM. Seven days after culture, the number of colonies (≥50 cells were counted) was counted under an inverted microscope [51].

### RNA Interference

Bim-speciﬁc and mock small interfering RNAs (siRNAs) were purchased from Dharmacon RNA Tech (Lafayette, CO). Transfection of anti-Bim or the control siRNA duplexes into EOL-1 cells involved use of the Cell Line Nucleofector Kit T (Amaxa, Gaithersburg, MD) and program O-17 according to the manufacturer's instructions [23,24]. Twenty-four h after siRNA transfection, the cells were exposed to drug treatment.

### Tumor xenograft experiments

Male nu/nu BALB/c mice were bred at the animal facility of Sun Yat-sen University. The mice were housed in barrier facilities with a 12-h light dark cycle, with food and water available *ad libitum*. BaF3-T674I cells (1×10^7^) resuspended in 100 μL of matrigel (Becton-Dickson Biosciences Pharmingen, San Jose, CA) were inoculated subcutaneously on the ﬂanks of 4- to 6-week-old male nude mice. Tumors were measured every other day with use of calipers. Tumor volumes were calculated by the following formula: a^2^×b×0.4, where *a* is the smallest diameter and *b* is the diameter perpendicular to a. S116836 was dissolved in 0.5% carboxymethylcellulose (CMC) (Sigma-Aldrich, Shanghai, China). Mice in the control group received 0.5% CMC. The body weight, feeding behavior and motor activity of each animal were monitored as indicators of general health. The animals were then euthanized, and tumor xenografts were immediately removed, weighed, stored and ﬁxed. All animal studies were conducted with the approval of the Sun Yat-sen University Institutional Animal Care and Use Committee.

### Immunohistochemical staining

Formalin-ﬁxed xenografts were embedded in parafﬁn and sectioned according to standard techniques. Tumor xenograft sections (4 μm) were immunostained using the MaxVision kit (Maixin Biol, Fuzhou, China) [23,24,46,49]. The primary antibody was rabbit anti-human Ki-67 (Maixin Biol, Fuzhou, China) without dilution. 50 μl MaxVision^TM^ reagent was applied to each slide. Color was developed with 0.05% diaminobenzidine and 0.03% H_2_O_2_ in 50 mM Tris–HCl (pH 7.6), and the slides were counterstained with hematoxylin. A negative control for Ki-67 was also included for each xenograft specimen by substituting the primary antibody with pre-immune rabbit serum.

### Pharmacokinetic study

Four Sprague–Dawley (SD) rats, male and female in half, weight 180-220 g, were fasted for 12 h before the initiation of the study. During the study they could eat food and drink water freely. All rats were given a dose of 25 mg/kg of S116836 in 0.5% carboxylmethylcellulose via oral gavage. Peripheral blood samples (approximately 0.3 mL per sample) were collected from each animal via the orbital venous plexus at 5, 15, 30 min, 1, 2, 3, 4, 6, 8, 12, 21, 24, 30, 36, 48, 60 and 72 h post-dose. Blood samples were centrifuged within 10 min of collection and plasma was harvested. Plasma samples were stored at 4 °C until analysis.

### Plasma sample preparation and LC-MS/MS analysis

To prepare the plasma sample for LC-MS/MS analysis (liquid chromatography coupled with tandem mass spectrometry), 150 μl internal standard solution (5 μg/mL, acetonitrile) was added to 50 μl rat plasma samples to precipitate proteins. After centrifugation for 30 min (13000 rpm, 4 °C), 20 μl of the supernatant was introduced into the LC-MS/MS system. Detection was carried out using a API 3000 mass spectrometer (Applied Biosystems, Foster City, CA) with TurboIonSpray source interface. The processed samples were injected on a CAPCELL PAK C_18_ column (2.0 mm × 50 mm, 5 μm; Shiseido, Japan). The system was run in isocratic mode with mobile phase consisting of methanol and water in the ratio of 90:10 (v/v) at a flow rate of 0.3 mL/min. An electrospray ion source in the negative-ion multiple reaction monitoring (MRM) mode was used for detection. The MRM transition channel was m/z 517.3 to m/z 233.2 for S116836 and m/z 260.0 to m/z 183.0 for internal standard (Propranolol, Sigma). Ionization temperature was set as 400 °C. Data acquisition and quantitation were performed using Analyst 1.4 (Applied Biosystems, MDS Sciex Toronto, Canada).

### Pharmacokinetic analysis

All pharmacokinetic parameters were calculated by DAS 2.0 (Clinical drug research center of Shanghai University of Traditional Chinese Medicine, Shanghai, China). Parameters are presented as a mean ± standard deviation (SD).

### Statistical analysis

All experiments were performed at least thrice, and results are expressed as mean ± 95% confidence interval (CI) unless otherwise stated. GraphPad Prism 5.0 software (GraphPad Software) was used for statistical analysis. Comparisons between two groups involved two-tailed Student's *t* test, and comparisons among multiple groups involved one-way ANOVA with *post hoc* intergroup comparison using Tukey test. A P value of <0.05 was considered statistically significant.

## SUPPLEMENTARY MATERIAL FIGURE



## References

[1] Rothenberg ME (1998). Eosinophilia. N Engl J Med.

[2] Gotlib J (2012). World Health Organization-defined eosinophilic disorders: 2012 update on diagnosis, risk stratification, and management. Am J Hematol.

[3] Valent P, Klion AD, Horny HP, Roufosse F, Gotlib J, Weller PF, Hellmann A, Metzgeroth G, Leiferman KM, Arock M, Butterfield JH, Sperr WR, Sotlar K, Vandenberghe P, Haferlach T, Simon HU, Reiter A, Gleich GJ (2012). Contemporary consensus proposal on criteria and classification of eosinophilic disorders and related syndromes. J Allergy Clin Immunol.

[4] Crane MM, Chang CM, Kobayashi MG, Weller PF (2010). Incidence of myeloproliferative hypereosinophilic syndrome in the United States and an estimate of all hypereosinophilic syndrome incidence. J Allergy Clin Immunol.

[5] Gotlib J, Cools J (2008). Five years since the discovery of FIP1L1-PDGFRA: what we have learned about the fusion and other molecularly defined eosinophilias. Leukemia.

[6] Montano-Almendras CP, Essaghir A, Schoemans H, Varis I, Noel LA, Velghe AI, Latinne D, Knoops L, Demoulin JB (2012). ETV6-PDGFRB and FIP1L1-PDGFRA stimulate human hematopoietic progenitor cell proliferation and differentiation into eosinophils: the role of nuclear factor-kappaB. Haematologica.

[7] Cools J, DeAngelo DJ, Gotlib J, Stover EH, Legare RD, Cortes J, Kutok J, Clark J, Galinsky I, Griffin JD, Cross NC, Tefferi A, Malone J, Alam R, Schrier SL, Schmid J, Rose M, Vandenberghe P, Verhoef G, Boogaerts M, Wlodarska I, Kantarjian H, Marynen P, Coutre SE, Stone R, Gilliland DG (2003). A tyrosine kinase created by fusion of the PDGFRA and FIP1L1 genes as a therapeutic target of imatinib in idiopathic hypereosinophilic syndrome. N Engl J Med.

[8] Hart TK, Cook RM, Zia-Amirhosseini P, Minthorn E, Sellers TS, Maleeff BE, Eustis S, Schwartz LW, Tsui P, Appelbaum ER, Martin EC, Bugelski PJ, Herzyk DJ (2001). Preclinical efficacy and safety of mepolizumab (SB-240563), a humanized monoclonal antibody to IL-5, in cynomolgus monkeys. J Allergy Clin Immunol.

[9] Spergel JM, Rothenberg ME, Collins MH, Furuta GT, Markowitz JE, Fuchs G, O'Gorman MA, Abonia JP, Young J, Henkel T, Wilkins HJ, Liacouras CA (2012). Reslizumab in children and adolescents with eosinophilic esophagitis: results of a double-blind, randomized, placebo-controlled trial. J Allergy Clin Immunol.

[10] Ghazi A, Trikha A, Calhoun WJ (2012). Benralizumab--a humanized mAb to IL-5Rα with enhanced antibody-dependent cell-mediated cytotoxicity--a novel approach for the treatment of asthma. Expert Opin Biol Ther.

[11] Giles FJ, Cortes JE, Kantarjian HM (2005). Targeting the kinase activity of the BCR-ABL fusion protein in patients with chronic myeloid leukemia. Curr Mol Med.

[12] Blagosklonny MV (2002). STI-571 must select for drug-resistant cells but ‘no cell breathes fire out of its nostrils like a dragon’. Leukemia.

[13] Blagosklonny MV (2004). Do cells need CDK2 and … Bcr-Abl?. Cell Death Differ.

[14] Gugliotta G, Castagnetti F, Palandri F, Baccarani M, Rosti G (2011). Imatinib in chronic myeloid leukemia elderly patients. Aging (Albany NY).

[15] Lierman E, Michaux L, Beullens E, Pierre P, Marynen P, Cools J, Vandenberghe P (2009). FIP1L1-PDGFRalpha D842V, a novel panresistant mutant, emerging after treatment of FIP1L1-PDGFRα T674I eosinophilic leukemia with single agent sorafenib. Leukemia.

[16] Cassuto O, Dufies M, Jacquel A, Robert G, Ginet C, Dubois A, Hamouda A, Puissant A, Luciano F, Karsenti JM, Legros L, Cassuto JP, Lenain P, Auberger P (2012). All tyrosine kinase inhibitor-resistant chronic myelogenous cells are highly sensitive to ponatinib. Oncotarget.

[17] Cerny-Reiterer S, Meyer RA, Herrmann H, Peter B, Gleixner KV, Stefanzl G, Hadzijusufovic E, Pickl WF, Sperr WR, Melo JV, Maeda H, Jager U, Valent P (2014). Identification of heat shock protein 32 (Hsp32) as a novel target in acute lymphoblastic leukemia. Oncotarget.

[18] Demidenko ZN, An WG, Lee JT, Romanova LY, McCubrey JA, Blagosklonny MV (2005). Kinase-addiction and bi-phasic sensitivity-resistance of Bcr-Abl- and Raf-1-expressing cells to imatinib and geldanamycin. Cancer Biol Ther.

[19] Pellicano F, Simara P, Sinclair A, Helgason GV, Copland M, Grant S, Holyoake TL (2011). The MEK inhibitor PD184352 enhances BMS-214662-induced apoptosis in CD34+ CML stem/progenitor cells. Leukemia.

[20] Verstovsek S, Giles FJ, Quintas-Cardama A, Manshouri T, Huynh L, Manley P, Cortes J, Tefferi A, Kantarjian H (2006). Activity of AMN107, a novel aminopyrimidine tyrosine kinase inhibitor, against human FIP1L1-PDGFR-alpha-expressing cells. Leuk Res.

[21] Pan J, Quintas-Cardama A, Manshouri T, Giles FJ, Lamb P, Tefferi A, Cortes J, Kantarjian H, Verstovsek S (2007). The novel tyrosine kinase inhibitor EXEL-0862 induces apoptosis in human FIP1L1-PDGFR-alpha-expressing cells through caspase-3-mediated cleavage of Mcl-1. Leukemia.

[22] Cools J, Stover EH, Boulton CL, Gotlib J, Legare RD, Amaral SM, Curley DP, Duclos N, Rowan R, Kutok JL, Lee BH, Williams IR, Coutre SE, Stone RM, DeAngelo DJ, Marynen P, Manley PW, Meyer T, Fabbro D, Neuberg D, Weisberg E, Griffin JD, Gilliland DG (2003). PKC412 overcomes resistance to imatinib in a murine model of FIP1L1-PDGFRα-induced myeloproliferative disease. Cancer Cell.

[23] Jin Y, Ding K, Li H, Xue M, Shi X, Wang C, Pan J (2014). Ponatinib efficiently kills imatinib-resistant chronic eosinophilic leukemia cells harboring gatekeeper mutant T674I FIP1L1-PDGFRα: roles of Mcl-1 and β-catenin. Mol Cancer.

[24] Shen Y, Shi X, Pan J (2013). The conformational control inhibitor of tyrosine kinases DCC-2036 is effective for imatinib-resistant cells expressing T674I FIP1L1-PDGFRα. PLoS One.

[25] Ren X, Pan X, Zhang Z, Wang D, Lu X, Li Y, Wen D, Long H, Luo J, Feng Y, Zhuang X, Zhang F, Liu J, Leng F, Lang X, Bai Y, She M, Tu Z, Pan J, Ding K (2013). Identification of GZD824 as an orally bioavailable inhibitor that targets phosphorylated and nonphosphorylated breakpoint cluster region-Abelson (Bcr-Abl) kinase and overcomes clinically acquired mutation-induced resistance against imatinib. J Med Chem.

[26] Bu Q, Cui L, Li J, Du X, Zou W, Ding K, Pan J (2014). SAHA and S116836, a novel tyrosine kinase inhibitor, synergistically induce apoptosis in imatinib-resistant chronic myelogenous leukemia cells. Cancer Biol Ther.

[27] Green DR, Kroemer G (2004). The pathophysiology of mitochondrial cell death. Science.

[28] Gordon PM, Fisher DE (2010). Role for the proapoptotic factor BIM in mediating imatinib-induced apoptosis in a c-KIT-dependent gastrointestinal stromal tumor cell line. J Biol Chem.

[29] Gong Y, Somwar R, Politi K, Balak M, Chmielecki J, Jiang X, Pao W (2007). Induction of BIM is essential for apoptosis triggered by EGFR kinase inhibitors in mutant EGFR-dependent lung adenocarcinomas. PLoS Med.

[30] O'Connor L, Strasser A, O'Reilly LA, Hausmann G, Adams JM, Cory S, Huang DC (1998). Bim: a novel member of the Bcl-2 family that promotes apoptosis. EMBO J.

[31] O'Reilly LA, Cullen L, Visvader J, Lindeman GJ, Print C, Bath ML, Huang DC, Strasser A (2000). The proapoptotic BH3-only protein bim is expressed in hematopoietic, epithelial, neuronal, and germ cells. Am J Pathol.

[32] Essafi A, Fernandez de Mattos S, Hassen YA, Soeiro I, Mufti GJ, Thomas NS, Medema RH, Lam EW (2005). Direct transcriptional regulation of Bim by FoxO3a mediates STI571-induced apoptosis in Bcr-Abl-expressing cells. Oncogene.

[33] Hubner A, Barrett T, Flavell RA, Davis RJ (2008). Multisite phosphorylation regulates Bim stability and apoptotic activity. Mol Cell.

[34] Qi XJ, Wildey GM, Howe PH (2006). Evidence that Ser87 of BimEL is phosphorylated by Akt and regulates BimEL apoptotic function. J Biol Chem.

[35] Luciano F, Jacquel A, Colosetti P, Herrant M, Cagnol S, Pages G, Auberger P (2003). Phosphorylation of Bim-EL by Erk1/2 on serine 69 promotes its degradation via the proteasome pathway and regulates its proapoptotic function. Oncogene.

[36] Salemi S, Yousefi S, Simon D, Schmid I, Moretti L, Scapozza L, Simon HU (2009). A novel FIP1L1-PDGFRA mutant destabilizing the inactive conformation of the kinase domain in chronic eosinophilic leukemia/hypereosinophilic syndrome. Allergy.

[37] Cartlidge RA, Thomas GR, Cagnol S, Jong KA, Molton SA, Finch AJ, McMahon M (2008). Oncogenic BRAF(V600E) inhibits BIM expression to promote melanoma cell survival. Pigment Cell Melanoma Res.

[38] Cragg MS, Jansen ES, Cook M, Harris C, Strasser A, Scott CL (2008). Treatment of B-RAF mutant human tumor cells with a MEK inhibitor requires Bim and is enhanced by a BH3 mimetic. J Clin Invest.

[39] Sheridan C, Brumatti G, Martin SJ (2008). Oncogenic B-RafV600E inhibits apoptosis and promotes ERK-dependent inactivation of Bad and Bim. J Biol Chem.

[40] Costa DB, Halmos B, Kumar A, Schumer ST, Huberman MS, Boggon TJ, Tenen DG, Kobayashi S (2007). BIM mediates EGFR tyrosine kinase inhibitor-induced apoptosis in lung cancers with oncogenic EGFR mutations. PLoS Med.

[41] Cragg MS, Kuroda J, Puthalakath H, Huang DC, Strasser A (2007). Gefitinib-induced killing of NSCLC cell lines expressing mutant EGFR requires BIM and can be enhanced by BH3 mimetics. PLoS Med.

[42] Deng J, Shimamura T, Perera S, Carlson NE, Cai D, Shapiro GI, Wong KK, Letai A (2007). Proapoptotic BH3-only BCL-2 family protein BIM connects death signaling from epidermal growth factor receptor inhibition to the mitochondrion. Cancer Res.

[43] Han J, Goldstein LA, Gastman BR, Rabinowich H (2006). Interrelated roles for Mcl-1 and BIM in regulation of TRAIL-mediated mitochondrial apoptosis. J Biol Chem.

[44] Herrant M, Jacquel A, Marchetti S, Belhacene N, Colosetti P, Luciano F, Auberger P (2004). Cleavage of Mcl-1 by caspases impaired its ability to counteract Bim-induced apoptosis. Oncogene.

[45] Gillings AS, Balmanno K, Wiggins CM, Johnson M, Cook SJ (2009). Apoptosis and autophagy: BIM as a mediator of tumour cell death in response to oncogene-targeted therapeutics. FEBS J.

[46] Wu Y, Chen C, Sun X, Shi X, Jin B, Ding K, Yeung SC, Pan J (2012). Cyclin-dependent kinase 7/9 inhibitor SNS-032 abrogates FIP1-like-1 platelet-derived growth factor receptor alpha and bcr-abl oncogene addiction in malignant hematologic cells. Clinical Cancer Res.

[47] Valent P, Gleich GJ, Reiter A, Roufosse F, Weller PF, Hellmann A, Metzgeroth G, Leiferman KM, Arock M, Sotlar K, Butterfield JH, Cerny-Reiterer S, Mayerhofer M, Vandenberghe P, Haferlach T, Bochner BS, Gotlib J, Horny HP, Simon HU, Klion AD (2012). Pathogenesis and classification of eosinophil disorders: a review of recent developments in the field. Expert Rev Hematol.

[48] Shi X, Jin Y, Cheng C, Zhang H, Zou W, Zheng Q, Lu Z, Chen Q, Lai Y, Pan J (2009). Triptolide inhibits Bcr-Abl transcription and induces apoptosis in STI571-resistant chronic myelogenous leukemia cells harboring T315I mutation. Clinical Cancer Res.

[49] Jin Y, Lu Z, Ding K, Li J, Du X, Chen C, Sun X, Wu Y, Zhou J, Pan J (2010). Antineoplastic mechanisms of niclosamide in acute myelogenous leukemia stem cells: inactivation of the NF-kB pathway and generation of reactive oxygen species. Cancer Res.

[50] Pan J, Quintas-Cardama A, Kantarjian HM, Akin C, Manshouri T, Lamb P, Cortes JE, Tefferi A, Giles FJ, Verstovsek S (2007). EXEL-0862, a novel tyrosine kinase inhibitor, induces apoptosis in vitro and ex vivo in human mast cells expressing the KIT D816V mutation. Blood.

[51] Freund D, Oswald J, Feldmann S, Ehninger G, Corbeil D, Bornhauser M (2006). Comparative analysis of proliferative potential and clonogenicity of MACS-immunomagnetic isolated CD34+ and CD133+ blood stem cells derived from a single donor. Cell Prolif.

