# Supplementary material for: Antitumor activity of S116836, a novel tyrosine kinase inhibitor, against imatinib-resistant FIP1L1-PDGFRα-expressing cells
Source: Oncotarget. 2014 Jun 11;5(21):10407–20. doi: 10.18632/oncotarget.2090 (PMC4279382; doi:10.18632/oncotarget.2090)
Supplement: Supplementary file 1 [file oncotarget-05-10407-s001.pdf]

Antitumor activity of S116836, a novel tyrosine kinase inhibitor, against imatinib-resistant FIP1L1-PDGFRα-expressing cells

Supplementary Material

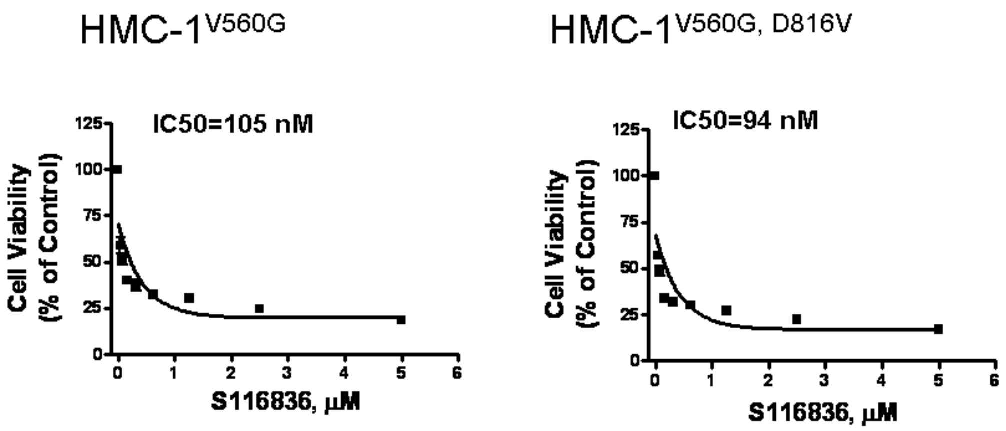

Figure S1. HMC-1^{V560G} and HMC-1^{V560G,D816V} cells were exposed escalating concentrations of S116836 for 72 hours, Cell viability was measured with MTS assay.
